# Snail-borne parasitic diseases: an update on global epidemiological distribution, transmission interruption and control methods

**DOI:** 10.1186/s40249-018-0414-7

**Published:** 2018-04-09

**Authors:** Xiao-Ting Lu, Qiu-Yun Gu, Yanin Limpanont, Lan-Gui Song, Zhong-Dao Wu, Kamolnetr Okanurak, Zhi-Yue Lv

**Affiliations:** 1 School of Public Health, Sun Yat-sen University, Guangzhou, 510080 China; 2 Faculty of Tropical Medicine, Mahidol University, Bangkok, 10400 Thailand; 3 Fifth Affiliated Hospital, Zhongshan School of Medicine, Sun Yat-sen University, Guangdong, China; 4 Key Laboratory of Tropical Disease Control (Sun Yat-sen University), Ministry of Education, Guangzhou, 510080 China; 5 Provincial Engineering Technology Research Center for Biological Vector Control, Guangzhou, 510080 China

**Keywords:** Snail-borne parasitic diseases, Epidemiology, Pathogenesis, Snail control

## Abstract

**Background:**

Snail-borne parasitic diseases, such as angiostrongyliasis, clonorchiasis, fascioliasis, fasciolopsiasis, opisthorchiasis, paragonimiasis and schistosomiasis, pose risks to human health and cause major socioeconomic problems in many tropical and sub-tropical countries. In this review we summarize the core roles of snails in the life cycles of the parasites they host, their clinical manifestations and disease distributions, as well as snail control methods.

**Main body:**

Snails have four roles in the life cycles of the parasites they host: as an intermediate host infected by the first-stage larvae, as the only intermediate host infected by miracidia, as the first intermediate host that ingests the parasite eggs are ingested, and as the first intermediate host penetrated by miracidia with or without the second intermediate host being an aquatic animal. Snail-borne parasitic diseases target many organs, such as the lungs, liver, biliary tract, intestines, brain and kidneys, leading to overactive immune responses, cancers, organ failure, infertility and even death. Developing countries in Africa, Asia and Latin America have the highest incidences of these diseases, while some endemic parasites have developed into worldwide epidemics through the global spread of snails. Physical, chemical and biological methods have been introduced to control the host snail populations to prevent disease.

**Conclusions:**

In this review, we summarize the roles of snails in the life cycles of the parasites they host, the worldwide distribution of parasite-transmitting snails, the epidemiology and pathogenesis of snail-transmitted parasitic diseases, and the existing snail control measures, which will contribute to further understanding the snail-parasite relationship and new strategies for controlling snail-borne parasitic diseases.

**Electronic supplementary material:**

The online version of this article (10.1186/s40249-018-0414-7) contains supplementary material, which is available to authorized users.

## Multilingual abstracts

Please see Additional file 1 for translations of the abstract into the five official working languages of the United Nations.

## Background

Snail-borne parasitic diseases (SBPDs) are major parasitic diseases that remain important public health issues worldwide, particularly in impoverished countries. Millions of people in approximately 90 countries have suffered from SBPDs, in which snails serve as the transmitting vectors and intermediate hosts (Table 1). Thus, the elimination or control of snails may be an alternative approach to the focused control of SBPDs and may effectively interrupt the transmission of SBPDs. Previous studies have documented the relationship between certain parasites and their intermediate host snails, but few studies have focused on the crucial importance of snails in the complex interactions between snails and snail-borne parasites [1]. Moreover, a better understanding of the basic biology of SBPDs and the vectors that transmit them are needed to explain the expanding geographical distribution of these diseases. This review discusses our current knowledge of SBPDs, with a particular focus on new evidence of the global distribution and the physical control of parasite-transmitting snails as well as the epidemiology and clinical aspects of SBPDs.

**Table 1:** The distribution of snails that can transmit parasitic diseases and the parasites they can carry

Categories	Distribution	*Ac*	*Cs*	*Fb*	*Fh*	*Of*	*Ov*	*Pw*	*Sh*	*Si*	*Sj*	*Smal*	*Sman*	*Smek*	References
Achatinidae															
*Achatina fulica*	East Africa, America, Brazil, China, Guam, India, Japan, Madagascar, Mauritius, Pacific Islands, Seychelles, Southeast Asia, Sri Lanka, Thailand, Zanzibar	+	–	–	–	–	–	–	–	–	–	–	–	–	[21, 22, 77]
Ampullariidae															
*Pila ampullacea*	Thailand	+	–	–	–	–	–	–	–	–	–	–	–	–	[21]
*Pi. angelica*	Thailand	+	–	–	–	–	–	–	–	–	–	–	–	–	[22]
*Pi. gracilis*	Thailand	+	–	–	–	–	–	–	–	–	–	–	–	–	[21]
*Pi. pesmei*	Thailand	+	–	–	–	–	–	–	–	–	–	–	–	–	[23]
*Pi. polita*	Thailand	+	–	–	–	–	–	–	–	–	–	–	–	–	[21]
*Pi. scutata*	Malaysia	+	–	–	–	–	–	–	–	–	–	–	–	–	[21]
*Pi. turbinis*	Thailand	+	–	–	–	–	–	–	–	–	–	–	–	–	[21]
*Pomacea canaliculata*	Argentina, Bolivia, Brazil, California, China, Dominican Republic, Florida, Guam, Hawaii, Indonesia, Japan, Korea, Laos, Malaysia, New Nyaya, North Korea, Paraguay, Philippines, Singapore, Sri Lanka, Texas, Thailand, Uruguay, Vietnam	+	–	–	–	–	–	–	–	–	–	–	–	–	[21, 77]
*Po. lineata*	Brazil	+	–	–	–	–	–	–	–	–	–	–	–	–	[3]
Ancylidae															
*Ferrissia tenuis*	India	+	–	–	–	–	–	–	–	–	–	–	–	–	[100]
Ariophantidae															
*Girasia peguensis*	China	+	–	–	–	–	–	–	–	–	–	–	–	–	[21]
*Hemiplecta distincta*	Thailand	+	–	–	–	–	–	–	–	–	–	–	–	–	[23]
*Microparmarion malayanus*	Burma, Malaysia	+	–	–	–	–	–	–	–	–	–	–	–	–	[21]
Assimineidae															
*Assiminea latericea*	China	+	–	–	–	–	–	–	–	–	–	–	–	–	[11]
Bithyniidae															
*Alocinma longicornis*	China	–	+	–	–	–	–	–	–	–	–	–	–	–	[11]
*Bithynia fuchsiania*	China	–	+	–	–	–	–	–	–	–	–	–	–	–	[12]
*Bit. funiculate*	Laos, Thailand	–	–	–	–	–	+	–	–	–	–	–	–	–	[13]
*Bit. goniompharus*	Laos, Thailand	–	–	–	–	–	+	–	–	–	–	–	–	–	[13]
*Bit. inflate*	–	–	–	–	–	+	–	–	–	–	–	–	–	–	–
*Bit. leachi*	Germany	+	–	–	–	+	–	–	–	–	–	–	–	–	[14]
*Bit. misella*	China	–	+	–	–	–	–	–	–	–	–	–	–	–	[12]
*Bit. siamensis*	Cambodia, Laos, Thailand	–	–	–	–	–	+	–	–	–	–	–	–	–	[13, 15]
*Bit. troscheli*	Russia	–	–	–	–	+	–	–	–	–	–	–	–	–	[16]
*Parafossarulus eximius*	China	+	–	–	–	–	–	–	–	–	–	–	–	–	[17]
*Pa. striatulus*	China	–	+	–	–	–	–	–	–	–	–	–	–	–	[12]
*Pa. sinensis*	China	–	+	–	–	–	–	–	–	–	–	–	–	–	[18]
*Pa. anomalospiralis*	China	–	+	–	–	–	–	–	–	–	–	–	–	–	[19]
*Pa. manchouricus*	China, Japan, Korea	–	+	–	–	–	–	–	–	–	–	–	–	–	[20]
Bradybaenida															
*Bradybaena despecta*	China, East Timor, Japan, Myanmar	+	–	–	–	–	–	–	–	–	–	–	–	–	[21]
*Br. ravida*	China, North Korea, Japan, Russia,	+	–	–	–	–	–	–	–	–	–	–	–	–	[21]
*Br. circulus*	Japan	+	–	–	–	–	–	–	–	–	–	–	–	–	[21]
*Br. similaris*	China, Brazil, East Timor, Japan, Pacific Islands	+	–	–	–	–	–	–	–	–	–	–	–	–	[21, 101]
*Euhadra quaesita*	Japan	+	–	–	–	–	–	–	–	–	–	–	–	–	[21]
*Plectotropis applanata*	China	+	–	–	–	–	–	–	–	–	–	–	–	–	[21]
Buccinidae															
*Clea helena*	Cambodia, Indonesia, Laos, Malaysia, Singapore, Thailand	+	–	–	–	–	–	–	–	–	–	–	–	–	[23, 102]
Camaenidae															
*Satsuma mercatoria*	Pacific Islands	+	–	–	–	–	–	–	–	–	–	–	–	–	[21]
*Camaena cicatricosa*	China, Japan, Myanmar, Pacific Islands, Vietnam	+	–	–	–	–	–	–	–	–	–	–	–	–	[21]
Cyclophoridae															
*Pupina complanata*	America, Malaysia	+	–	–	–	–	–	–	–	–	–	–	–	–	[21]
Helicarionidae															
*Parmarion martensi*	Japan, Hawaii	+	–	–	–	–	–	–	–	–	–	–	–	–	[103]
Lymnaeidae															
*Fossaria cubensis*	America, Bolivia, Caribbean Islands, Colombia, Cuba, Mexico, Uruguay, Venezuela	–	–	–	+	–	–	–	–	–	–	–	–	–	[43]
*Galba cousin*	Colombia, Ecuador, Venezuela	–	–	–	+	–	–	–	–	–	–	–	–	–	[43]
*G. glaticallsformis*	–	–	–	–	+	–	–	–	–	–	–	–	–	–	–
*G. pervia*	China	+	–	–	+	–	–	–	–	–	–	–	–	–	[44]
*G. truncatula*	Argentina, Bolivia, Brazil, Chile, Colombia, France, Italy, Mexico, Peru, Portugal, Spain, Switzerland, the Netherlands, Venezuela	+	–	–	+	–	–	–	–	–	–	–	–	–	[43, 45–47, 104, 105]
*Lymnaea bulimoides*	Mexico	–	–	–	+	–	–	–	–	–	–	–	–	–	[46]
*Ly. diaphana*	Argentina, Chile, Peru	–	–	–	+	–	–	–	–	–	–	–	–	–	[43]
*Ly. fuscus*	Sweden	–	–	–	+	–	–	–	–	–	–	–	–	–	[48]
*Ly. humilis*	Mexico	–	–	–	+	–	–	–	–	–	–	–	–	–	[46]
*Ly. japonica*	–	+	–	–	–	–	–	–	–	–	–	–	–	–	–
*Ly. neotropica*	Argentina, Peru	**–**	**–**	**–**	+	**–**	**–**	**–**	**–**	**–**	**–**	**–**	**–**	**–**	[43]
*Ly. obrussa*	Mexico	–	–	–	+	–	–	–	–	–	–	–	–	–	[46]
*Ly. ollula*	Japan, Korea	–	–	–	+	–	–	–	–	–	–	–	–	–	[49]
*Ly. palustris*	Sweden	+	–	–	+	–	–	–	–	–	–	–	–	–	[21, 48]
*Ly. rupestris*	Brazil	**–**	**–**	**–**	+	**–**	**–**	**–**	**–**	**–**	**–**	**–**	**–**	**–**	[43]
*Ly. tomentosa*	Australia	**–**	**–**	**–**	+	**–**	**–**	**–**	**–**	**–**	**–**	**–**	**–**	**–**	[50]
*Ly. viatrix*	Argentina, Bolivia, Brazil, Mexico, Peru, Uruguay	–	–	–	+	–	–	–	–	–	–	–	–	–	[43, 46]
*Ly. viridis*	Australia, China, Korea, Vietnam	**–**	**–**	**–**	+	**–**	**–**	**–**	**–**	**–**	**–**	**–**	**–**	**–**	[49, 50]
*Omphiscola glabra*	France, Germany, Italy	–	–	–	+	–	–	–	–	–	–	–	–	–	[45, 47]
*Pseudosuccinea columella*	Africa, Australia, Caribbean Islands, Central America, Europe, New Zealand, North America, South America, Tahiti	–	–	–	+	–	–	–	–	–	–	–	–	–	[10, 51]
*Radix auricularia*	China, Czech Republic, France, Germany, Iceland, Italy, Korea, Poland,	+	–	–	+	–	–	–	–	–	–	–	–	–	[11, 47, 52, 53]
*Ra. lagotis*	Austria, China, Czech Republic	+	–	–	+	–	–	–	–	–	–	–	–	–	[11, 45]
*Ra. natalensis*	Egypt, Senegal	+	–	–	+	–	–	–	–	–	–	–	–	–	[54]
*Ra. ovata (Ra. peregra)*	Czech Republic, France, Iceland, Italy, Poland, Spain, the Netherlands	+	–	–	+	–	–	–	–	–	–	–	–	–	[45, 47, 53]
*Ra. plicatula*	–	+	–	–	+	–	–	–	–	–	–	–	–	–	–
*Ra. swinhoei*	China, Japan, Poland, Thailand, Vietnam	–	–	–	+	–	–	–	–	–	–	–	–	–	[55]
*Stagnicola palustris*	Italy	–	–	–	+	–	–	–	–	–	**–**	**–**	**–**	**–**	[47]
Physidae															
*Physa acuta*	Japan, Peru	+	–	–	–	–	–	–	–	–	–	–	–	–	[21, 106]
Planorbidae															
*Biomphalaria alexandrina*	Egypt, Libya, Sudan	–	–	–	–	–	–	–	–	–	–	–	+	–	[24]
*Bio. amazonica*	–	–	–	–	–	–	–	–	–	–	–	–	+	–	–
*Bio. andecola*	–	–	–	–	–	–	–	–	–	–	–	–	+	–	–
*Bio. arabica*	Saudi Arabia	–	–	–	–	–	–	–	–	–	–	–	+	–	[25]
*Bio. camerunensis*	Cameroon	–	–	–	–	–	–	–	–	–	–	–	+	–	[26]
*Bio. choanomphala*	Albert, Kyoga, Victoria	–	–	–	–	–	–	–	–	–	–	–	+	–	[27]
*Bio. glabrata*	Caribbean Islands, south America	–	–	–	–	–	–	–	–	–	–	–	+	–	[24]
*Bio. helophila*	Cuba, Peru	+	–	–	–	–	–	–	–	–	–	–	+	–	[28, 106]
*Bio. intermedia*	–	–	–	–	–	–	–	–	–	–	–	–	+	–	–
*Bio. kuhniana*	China, Venezuela	–	–	–	–	–	–	–	–	–	–	–	+	–	[29]
*Bio. obstructa*	Cuba	–	–	–	–	–	–	–	–	–	–	–	+	–	[28]
*Bio. occidentalis*	–	–	–	–	–	–	–	–	–	–	–	–	+	–	–
*Bio. peregrine*	–	–	–	–	–	–	–	–	–	–	–	–	+	–	–
*Bio. pfeiffei*	Africa, Chad	–	–	–	–	–	–	–	–	–	–	–	+	–	[30]
*Bio. prona*	–	–	–	–	–	–	–	–	–	–	–	–	+	–	–
*Bio. schrommi*	–	–	–	–	–	–	–	–	–	–	–	–	+	–	–
*Bio. smithi*	Lake Edward	–	–	–	–	–	–	–	–	–	–	–	+	–	[27]
*Bio. stanleyi*	Lake Albert	–	–	–	–	–	–	–	–	–	–	–	+	–	[27]
*Bio. straminea*	Argentina, Brazil, Caribbean, China, Grenada, Guadeloupe, Martinique, Paraguay, St Lucia, Uruguay	–	–	–	–	–	–	–	–	–	–	–	+	–	[29]
*Bio. sudanica*	lakes and rivers through central and eastern Africa	–	–	–	–	–	–	–	–	–	–	–	+	–	[31]
*Bio. temascalensis*	–	–	–	–	–	–	–	–	–	–	–	–	+	–	–
*Bio. tenagophila*	Brazil	–	–	–	–	–	–	–	–	–	–	–	+	–	[29]
*Bulinus africanus*	Kenya	–	–	–	–	–	–	–	+	–	–	–	–	–	[32]
*Bu. bavayi*	Madagascar	–	–	–	–	–	–	–	+	–	–	–	–	–	[33]
*Bu. beccari*	Saudi Arabia	–	–	–	–	–	–	–	+	–	–	–	–	–	[25]
*Bu. camerunensis*	Cameroon	–	–	–	–	–	–	–	+	–	–	–	–	–	[34]
*Bu. contortus*	Portugal	–	–	–	–	–	–	–	+	–	–	–	–	–	[35]
*Bu. crystallinus*	–	–	–	–	–	–	–	–	–	+	–	–	–	–	–
*Bu. forakalii*	Cameroon, Chad, Gabon, Rhodesia, Senegal, Tanzania, Zaire	–	–	–	–	–	–	–	+	+	–	–	–	–	[25, 30, 36]
*Bu. globosus*	Cameroon, Kenya, Lake Victoria area, Nigeria, Pemba, Senegal, Unguja Island, Zanzibar	–	–	–	–	–	–	–	+	+	–	–	–	–	[32, 34, 37, 38]
*Bu. liratus*	Madagascar	–	–	–	–	–	–	–	+	–	–	–	–	–	[33]
*Bu. nasutus*	Kenya, Zanzibar	–	–	–	–	–	–	–	+	–	–	–	–	–	[32, 37]
*Bu. nyassanus*	Denmark, Malawi	–	–	–	–	–	–	–	+	–	–	–	–	–	[39]
*Bu. obtusispira*	Madagascar	–	–	–	–	–	–	–	+	–	–	–	–	–	[33]
*Bu. reticulatus*	Cameroon	–	–	–	–	–	–	–	–	+	–	–	–	–	[37]
*Bu. rohlfsi*	Nigeria	–	–	–	–	–	–	–	+	–	–	–	–	–	[38]
*Bu. senegalensis*	Cameroon, Senegal	–	–	–	–	–	–	–	+	–	–	–	–	–	[34]
*Bu. tropicus*	Cameroon	–	–	–	–	–	–	–	+	–	–	–	–	–	[34]
*Bu. truncatus*	Cameroon, Chad, Egypt, Nile Delta, North Africa, Portugal, Saudi Arabia, Senegal, Sub-Saharan Africa, Sudan	–	–	–	–	–	–	–	+	+	–	–	–	–	[25, 30, 34, 35]
*Bu. ugandae*	Lake Victoria	–	–	–	–	–	–	–	+	–	–	–	–	–	[32]
*Bu. umbilicatus*	Senegal	–	–	–	–	–	–	–	+	–	–	–	–	–	[34]
*Bu. wright*	Saudi Arabia	–	–	–	–	–	–	–	+	–	–	–	–	–	[25]
*Gyraulus convexiusculus*	China, India, Korea, Thailand	+	–	+	–	–	–	–	–	–	–	–	–	–	[11, 22, 40, 41]
*Hippeutis cantori*	China, Korea	+	–	+	–	–	–	–	–	–	–	–	–	–	[11, 41]
*H. umbilicalis*	Bangladesh, China, Thailand	+	–	+	–	–	–	–	–	–	–	–	–	–	[11, 42]
*Indoplanorbis exustus*	Camroon, Malaysia, Thailand	+	–	–	–	–	–	–	–	–	–	–	–	–	[21, 22, 107]
*Lanistes carinatus*	Thailand	+	–	–	–	–	–	–	–	–	–	–	–	–	[23]
*La. purpureus*	Kenya	–	–	–	–	–	–	–	+	–	–	–	–	–	[32]
*Planorbarius metidjensis*	Portugal	–	–	–	–	–	–	–	+	–	–	–	–	–	[35]
*Segmentina hemisphaerula*	Korea, Thailand	+	–	+	–	–	–	–	–	–	–	–	–	–	[41]
*Seg. trochoideus*	Bangladesh, Thailand	–	–	+	–	–	–	–	–	–	–	–	–	–	[42]
Pleuroseridae															
*Semisulcospira amurensis*	–	–	–	–	–	–	–	+	–	–	–	–	–	–	–
*Sem. cancellata*	China	+	+	–	–	–	–	+	–	–	–	–	–	–	[108]
*Sem. kurodai*	–	–	–	–	–	–	–	+	–	–	–	–	–	–	–
*Sem. libertina*	China	–	–	–	–	–	–	+	–	–	–	–	–	–	[109]
*Sem. mandarina*	–	–	–	–	–	–	–	+	–	–	–	–	–	–	–
*Sem. peregrinorum*	–	–	–	–	–	–	–	+	–	–	–	–	–	–	–
*Sem. toucheana*	–	–	–	–	–	–	–	+	–	–	–	–	–	–	–
Pomatiopsidae															
*Neotricula aperta*	Cambodia, Laos, Thailand	–	–	–	–	–	–	–	–	–	–	–	–	+	[59]
*Oncomelania hupensis*	China, Indonesia, Philippines	+	–	–	–	–	–	–	–	–	+	–	–	–	[60]
*Robertsiella kaporensis*	Malaysia	–	–	–	–	–	–	–	–	–	–	+	–	–	[110]
Subulinidae															
*Allopeas kyotoensis*	Japan	+	–	–	–	–	–	–	–	–	–	–	–	–	[21]
*Opeas javanicum*	Pacific Islands	+	–	–	–	–	–	–	–	–	–	–	–	–	[21]
*Subulina octona*	Brazil, Pacific Islands	+	–	–	–	–	–	–	–	–	–	–	–	–	[21, 111]
Succineidae															
*Succinea lauta*	Japan	+	–	–	–	–	–	–	–	–	–	–	–	–	[21]
*Su. pfeifferi*	Norway	+	–	–	–	–	–	–	–	–	–	–	–	–	[112]
Thiaridae															
*Melanoides tuberculata*	America, Australia, Brazil, China, Egypt, India, Iran, Israel, Jordan, Kenya, Mexico, Saudi Arabia, Thailand, United Arab Emirates, Venezuela	–	+	–	–	–	–	+	+	–	–	–	–	–	[22, 32, 56, 57]
*Tarebia granifera (M. granifera)*	South-East Asia, North and South America and Africa	–	–	–	–	–	–	+	–	–	–	–	–	–	[58]
Viviparidae															
*Bellamya aeruginosa*	China	+	–	–	–	–	–	–	–	–	–	–	–	–	[21]
*Be. ingallsiana*	Malaysia	+	–	–	–	–	–	–	–	–	–	–	–	–	[21]
*Be. quadrata*	China	+	–	–	–	–	–	–	–	–	–	–	–	–	[113]
*Cipangopaludina chinensis*	China, Japan, North Korea	+	–	–	–	–	–	–	–	–	–	–	–	–	[21]
*Filopaludina martensi martensi*	Thailand	+	–	–	–	–	–	–	–	–	–	–	–	–	[23]
*F. sumatrensis polygramma*	Thailand	+	–	–	–	–	–	–	–	–	–	–	–	–	[23]
*Sinotaia quadrata*	Japan	+	–	–	–	–	–	–	–	–	–	–	–	–	[114]

*Ac = Angiostrongylus cantonensis*; *Cs = Clonorchis sinensis*; *Fb = Fasciolopsis buski*; *Fh = Fasciola hepatica*; *Of = Opisthorchis felineus*; *Ov = Opisthorchis viverrini*; *Pw = Paragonimus westermani*; *Sh = Schistosoma haematobium; Si = Schistosoma intercalatum; Sj = Schistosoma japonicum*; *Smal = Schistosoma malayensis*; *Sman = Schistosoma mansoni*; *Smek = Schistosoma mekongi*

### Roles of snails in the life cycles of parasites

**Fig. 1:**
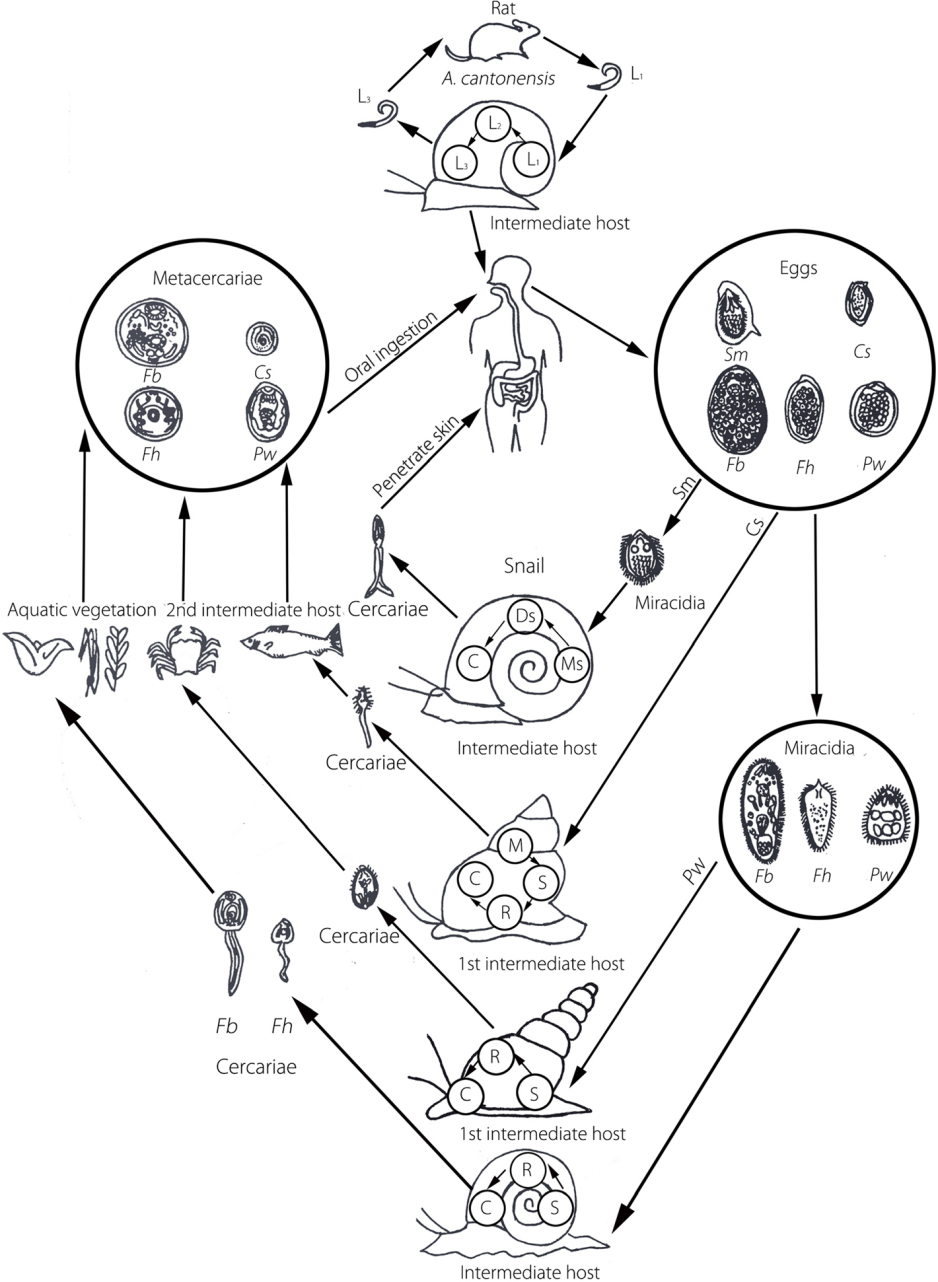
Schematic illustration of the life cycles of six snail-borne parasites, including *A. cantonensis*, *C. sinensis*, *F. buski*, *F. hepatica, P. westermani* and *S. mansoni.* C: Ceceariae; *Cs*: *Clonorchis sinensis*; Ds: Daughter sporocysts; *Fb*: *Fasciolopsis buski*; *Fh*: *Fasciola hepatica*; L1: first-stage larvae; L2: second-stage larvae; L3: third-stage larvae; M: Miracidia; Ms.: Mother sporocysts; *Pw*: *Paragonimus westermani*; R: Rediae; S: Sporocysts; *Sm*: *Schistosoma mansoni*

Based on the roles of snails and the developmental stages of the parasites they host, SBPDs can be divided into five groups (Fig. 1). Group I includes Nematoda diseases in which snails act as an intermediate host, a representative pathogen for which is *Angiostrongylus cantonensis.* The first-stage larvae (L1) of *A. cantonensis* are shed into the external environment via rat faeces (definitive host) [2]. The snails become infected when they ingest the infected rat faeces or when these larvae penetrate their body wall or respiratory pores [3]. L1 moult twice into second-stage (L2) and third-stage larvae (L3) in the mollusc tissue [3]. The other four groups are associated with Trematoda. In group II, snails serve as the only intermediate host and become infected by penetrating miracidia. A typical example of a group II SBPD is *Schistosoma mansoni*. The eggs of the parasite hatch and release ciliated miracidia that penetrate the snails and asexually replicate through two sporocyst generations (mother and daughter sporocyst stages). Finally, thousands of cercariae are shed into the water, that infect humans who come into contact with the contaminated water [4]. In group III, snails are the first intermediate hosts and become infected by ingesting parasite eggs. *Clonorchis sinensis* is a typical species of this group. In these parasites, after miracidia are released from the eggs they subsequently develop into sporocysts and finally form cercariae that then infect freshwater fish, which are the second intermediate host [5]. In group IV, snails may become the first intermediate host and are infected by miracidia [6]. For example, *Paragonimus westermani* eggs hatch and release miracidia into the water, which undergo various stages within the snails. The miracidia develop into sporocysts, rediae and cercariae successively, then invade a second intermediate host, such as crabs and crayfish [6]. In group V, snails are the first intermediate host and are infected by penetrating miracidia, with the second intermediate host being aquatic plants [7, 8], such as *Fasciolopsis buski* and *F. hepatica*. The eggs hatch into ciliated miracidia that swim to snails such as *P. westermani* [9]. After invading the snails, they transform into sporocysts, rediae, and then cercariae that encyst on aquatic vegetation and become metacercariae [7, 8, 10].

In summary, snails are the only intermediate hosts of *A. cantonensis* and *S. mansoni,* while they serve as the first intermediate hosts of *C. sinensis, P. westermani, F. buski*, and *F. hepatica*. The parasites undergo several developmental stages within the snails, demonstrating the vital role of snails in SBPDs (Fig. 1).

### Global distribution of parasite-transmitting snails

Terrestrial and freshwater snails are intermediate hosts in the life cycles of various parasites. The distributions of 136 snail species from 18 families are described in Table 1.

Bithyniidae snails are intermediate hosts of *C. sinensis*, *Opisthorchis felineus* and *O. viverrini* and are endemic to several geographical regions in Asia and Europe, including Cambodia, China, Germany, Japan, Korea, Laos, Russia and Thailand [11–20]. Planorbidae snails are the intermediate hosts of *F. buski*, *Schistosoma haematobium*, *S. intercalatum* and *S. mansoni*. These snails are widespread throughout Africa, Asia and Latin America and serve as intermediate hosts of *F. hepatica* [11, 21–42]. Lymnaeidae snails are primarily found in Africa, Asia, North America and South America [10, 11, 21, 43–55]. Thiaridae snails, which are reported to serve as intermediate hosts for many parasites, such as *P. westermani, C. sinensis* and *S. haematobium*, are distributed worldwide, but primarily in Africa, Asia, Oceania, North America and South America [22, 32, 56–58] (Table 1).

Most parasites require a specific snail species as an intermediate host. For example, the life cycles of *Schistosoma japonicum* and *S. mekongi* require *Oncomelania hupensis* and *Neotricula aperta* as their intermediate hosts, respectively. These snails have limited distributions: *N. aperta* is endemic to Cambodia, Laos and Thailand [59], and *O. hupensis* is found only in China, Indonesia and the Philippines [60]. *Pomacea canaliculata*, which is native to South America, was introduced to China in the 1980s and has since replaced *Achatina fulica* to become a major intermediate host that is the primary cause of *A. cantonensis* infection in humans in China [61].

To some extent, a correlation exists between the distribution of snails and parasitic diseases. Mapping the distribution of snails may help clarify their interactions with parasitic diseases and identify environmental factors that will help better detect and predicting the prevalence of these diseases. Geographic information systems (GISs) and remote sensing (RS) techniques have been increasingly used to map and model the distribution of snails. These techniques, which provide information on snail habitats and dispersal areas and to predict snail-infested regions, have been utilized masterfully in several areas, including Africa [62]. Spatial-temporal scan statistics, another new technique, accurately detects snail-infested areas to determine targeted intervention and surveillance strategies [63].

### Epidemiology and pathogenesis of snail-transmitted parasitic diseases

#### Paragonimiasis

**Fig. 2:**
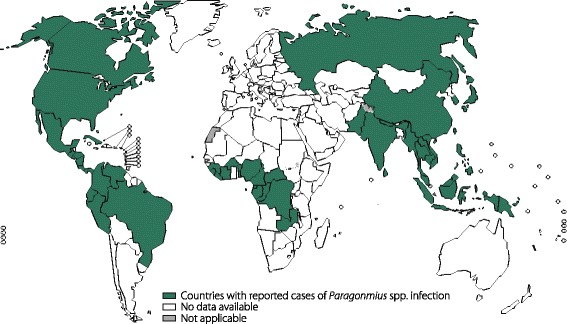
Global distribution of paragonimiasis

Paragonimiasis, which is caused by members of the genus *Paragonimus*, is an inflammatory lung disease. Approximately 20 million people are infected with *Paragonimus* species (World Health Organization 2002) [64], and 293 million are at risk of infection [65]. The disease is primarily endemic to China, Korea, and Japan, as well as several other Asian countries [66]. *P. westermani* is the most common and widespread species of this genus and is widely distributed in Asia (Fig. 2). This parasite can infect human lungs, brain, spinal cord, and other organs, causing pulmonary, neurological, and abdominal diseases [66].

#### Fasciolopsiasis

**Fig. 3:**
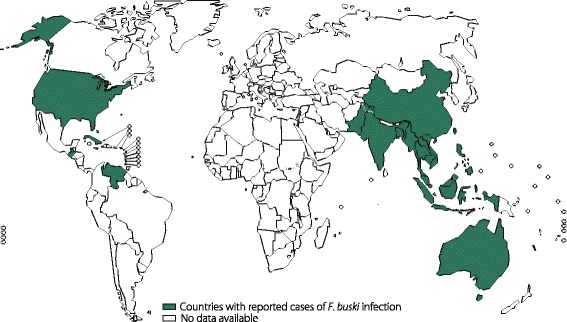
Global distribution of fasciolopsiasis

Fasciolopsiasis, which results from *F. buski* infection, is highly prevalent in Asian countries and can be fatal in endemic areas [9] (Fig. 3). Generally, low-intensity *F. buski* infections cause mild symptoms, such as diarrhoea, abdominal pain, and headaches. However, high-intensity infections can cause death due to extensive intestinal erosion, ulceration, haemorrhaging, abscesses, and inflammation [67].

#### Clonorchiasis and opisthorchiasis

**Fig. 4:**
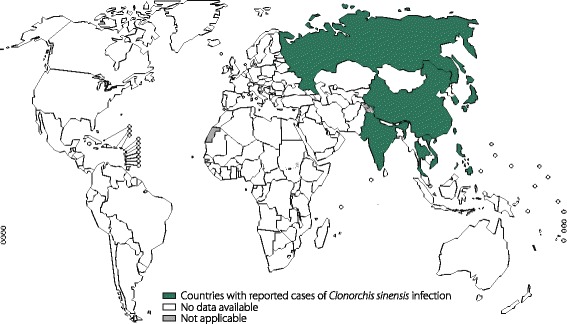
Global distribution of clonorchiasis

**Fig. 5:**
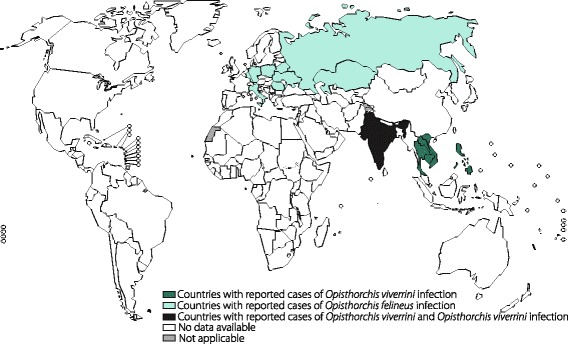
Global distribution of opisthorchiasis

Pathogens that cause clonorchiasis and opisthorchiasis include the liver flukes *C. sinensis*, *O. viverrini* and *O. felineus*, members of the Opisthorchiidae family. Thirty-five million people are estimated to be infected with *C. sinensis* worldwide, approximately 15 million of whom are Chinese (Fig. 4). Approximately 10 million people are infected with *O. viverrini*, with 4 in 5 infections having occurred in Thailand and the remainder having occurred in Laos [68]. It is believed that 1.2 million people are infected with *O. felineus*, which is endemic to the area encompassing the former Soviet Union [67] (Fig. 5). *C. sinensis* has been classified by the International Agency for Research on Cancer (IARC) as a probable carcinogen (group 2A), while *O. viverrini* has been definitively validated as a carcinogen (class 1) [69]. Patients with mild *C. sinensis* infections are generally asymptomatic or have few clinical manifestations (such as diarrhoea and abdominal pain) [67], while severe infections can lead to acute pain in the right upper abdomen. Patients carrying *O. viverrini* are typically asymptomatic. Severe opisthorchiasis can lead to obstructive jaundice, cirrhosis, cholangitis, acalculous cholecystitis, or bile peritonitis [70]. Acute *O. felineus* infections produce fever and hepatitis-like symptoms, while chronic infections results in obstruction, inflammation and fibrosis of the biliary tract, liver abscesses, pancreatitis, and suppurative cholangitis [71].

#### Fascioliasis

**Fig. 6:**
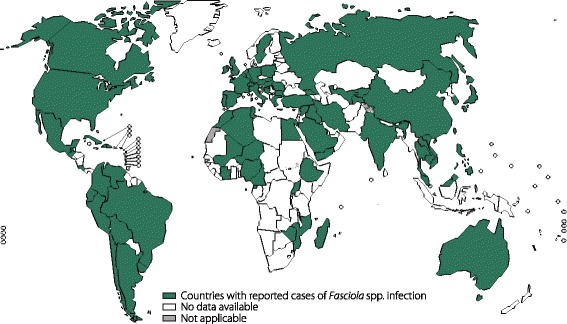
Global distribution of fascioliasis

Fascioliasis is a disease caused by the liver trematode, *F. hepatica*, and is responsible for zoonotic diseases, especially livestock [72]. Fascioliasis has historically been endemic in Andean countries, the Caribbean, the Caspian region, northern Africa and western Europe [10]; however, it has recently spread globally, including to many countries in Africa, the Americas, Asia, Europe and Oceania [1] (Fig. 6).

Fascioliasis manifests as intrahepatic and ectopic fascioliasis, with intrahepatic fascioliasis including acute and chronic phases. In the acute phase, which is caused by the migration of the immature trematode to the liver, clinical manifestations include fever, vomiting, abdominal pain, diarrhoea, urticaria, hepatomegaly and eosinophilia. During the chronic phase, when the flukes localize to the bile duct, the symptoms can present as intermittent biliary obstruction and inflammation [1, 67]. The migration of the parasites to other organs, such as the gastrointestinal tract, lungs, brain, muscles and eyes, results in ectopic fascioliasis without specific symptoms [73]. In recent years, fascioliasis has become a significant public health problem, causing extensive human morbidity (over 20 million cases reported worldwide) and considerable economic loss [43].

#### Angiostrongyliasis

**Fig. 7:**
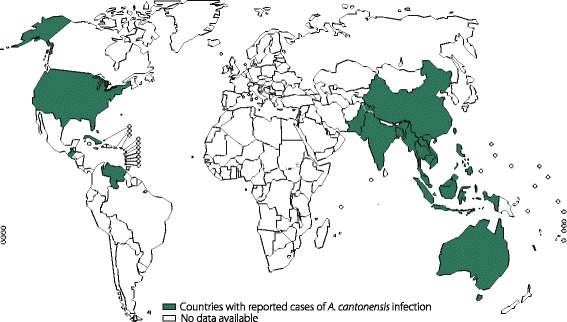
Global distribution of angiostrongyliasis. The figure was drawn according to integrated information from previous studies. Countries with reported disease cases are coloured green, and countries with no available data are coloured white

Angiostrongyliasis is caused by the emerging pathogen, *A. cantonensis*, which was first discovered in 1935 in Canton, China, by Chen [3]. Now Angiostrongyliasis has spread from endemic areas in the Pacific Basin and Southeast Asia to countries in the Americas, including Brazil, the Caribbean Islands and the USA, and has been found in many areas worldwide [74] (Fig. 7). By 2008, more than 2800 cases had been documented in nearly 300 countries and regions [61], of which the major outbreaks were reported in endemic areas, particularly in China. For example, an extensive outbreak of 160 cases that occurred in 2006 in Beijing, China, attracted a great deal of public attention [75]. Additionally, sporadic cases have been reported in Europe, primarily from travellers returning from endemic regions [61].

The primary clinical manifestations of human angiostrongyliasis, which is one type of larva migrans [74], include eosinophilic meningitis (EM), meningoencephalitis and ocular angiostrongyliasis (OA), among which, EM is the most common presentation in humans when the larvae migrate to the brain. [2]. Major symptoms of angiostrongyliasis include vomiting, nausea, paraesthesia, headaches and neck stiffness [61]. Severe EM and meningoencephalitis are also reported to lead to neurologic dysfunction, coma and even death in some cases [76]. When the larvae migrate to the host’s eyes, which is rare, the disease manifests as OA, with symptoms including diplopia, strabismus and vision loss ranging from blurred vision to blindness [77].

#### Schistosomiasis

**Fig. 8:**
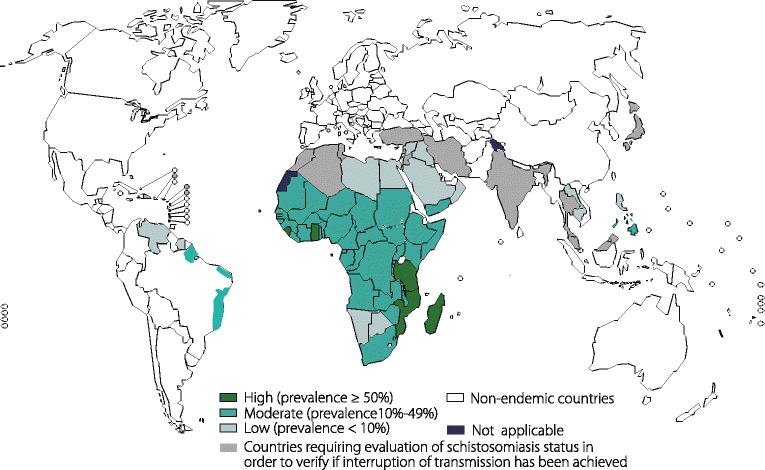
Global distribution of schistosomiasis. Figures 2, 3, 4, 5, 6 and 8, were obtained from the World Health Organization (WHO) at http://www.who.int/neglected_diseases/NTD__A_statistical_update_latest_data_available.pdf and were authorized by the WHO to reproduce in this review

Schistosomiasis, a neglected tropical disease, is an infection of blood flukes from the genus *Schistosoma* and has been reported in 78 countries in Africa, Asia and Latin America, especially in impoverished communities without access to a sound public health system [60, 78]. Schistosomiasis affects at least 230 million people worldwide, resulting in extensive social and economic burdens [4] (Fig. 8).

Schistosomiasis is an immune disease in which the body’s immune system overreacts to the eggs, cercariae, schistosomula and adult worms, leading to egg granulomas, cercarial dermatitis, vasculitis and endophlebitis, respectively [4].

Acute schistosomiasis occurs in individuals who are infected for the first time, presenting as fever, headache, abdominal pain, myalgia, malaise, fatigue and eosinophilia. Chronic schistosomiasis, which is common in endemic regions, manifests as non-specific intermittent rectal bleeding, abdominal pain and diarrhoea, heavily affecting people’s ability to study and work and can even lead to death [4].

In addition, *S. haematobium* is the only urogenital schistosomiasis pathogen whose typical symptom is haematuria [79]. Urogenital schistosomiasis may induce genital lesions, vaginal bleeding, pain during sexual intercourse, vulva nodules, and pathology of the prostate, seminal vesicles and other organs, with infertility being a potential long-term consequence [78].

In addition to the wide geographical distribution of SBPDs mentioned above, other SBPDs are distributed over a comparatively smaller scale. For example, echinostomiasis is primarily endemic in Southeast Asia, the Middle East and East Africa [80]. Halzoun, which is acquired by consuming raw freshwater fish containing *Clinostomum* metacercariae, has been reported in Japan, Korea, India and China [81].

#### Snail control

Intermediate hosts of various parasite species are essential in the transmission of SBPDs; Thus, the control of snail populations below a certain threshold is an efficient measure to limit the spread of SBPDs. Such control methods can be categorized into physical, chemical and biological measures.

Physical control measures aim at reducing snail populations through environmental management. For example, eliminating natural water bodies (such as marshes and ponds) and regulating human settlement in areas of risk are efficient measures. In some areas, proper drainage and environmental engineering have also decreased *S. haematobium* and *S. japonicum* transmission [82]. Another effective measure, mechanical disturbance, can potentially eliminate most snails by disturbing their epilithic habitats using boat-mounted rototillers or tractors and rakes. In addition, the removal of bird roosting sites, implementation of mechanized farming and the rotation of aquatic and xeromorphic crops can also reduce snail populations [83].

Chemical control generally involves the use of either synthetic or natural chemical molluscicides, and the application of chemical molluscicides remains one of the most efficient methods of snail control [84]. Copper sulfate, sodium pentachlorophenate (NaPCP), N-tritylmorpholine, and niclosamide (Bayluscide) were widely used from the 1950s to 1970s to control snails, especially to control schistosomiasis in Asia, Africa and South America [85]. In China, over 2000 chemicals have been developed and used since the 1950s, such as NaPCP, nicotinanilide, and bromoacetamide [84]. Among these synthetic molluscicides, only niclosamide is recommended by the World Health Organization; therefore, a 50% wettable powder of niclosamide ethanolamine salt (WPN) is the only synthetic compound available in China, where it has been widely used in snail control [84]. Remarkably, no clear evidence has emerged regarding snail resistance after extensive and prolonged niclosamide application for over 20 years [86] despite WPN being both toxic to fish and costly [84]. To address these problems, a novel molluscicide, quinoid-2′, 5-dichloro-4′-nitrosalicylanilide salt, has been developed that has the same molluscicidal effects as WPN but is cheaper and is significantly less toxic to fish [84]. Another new molluscicide, a niclosamide suspension concentrate, is physically more stable, more effective, and less toxic than WPN [87]. These molluscicides can be more useful than other snail control methods in areas endemic for schistosomiasis [84, 87].

Due to the high cost, toxicity, environmental contamination, and possible development of snail resistance to chemical molluscicides [88], natural molluscicides are rapidly being developed. Many plant extracts are potential molluscicides that are environmentally friendly, less toxic and are less likely to cause snails to develop resistance [89]. Many plant products have shown to be effective. For example, solvent extracts of fresh, mature *Solanum nigrum* leaves and species of the genus *Atriplex* repel *Biomphalaria alexandrina* [89, 90], while *Atriplex inflata* has been reported to repel *Galba truncatula* [90]. Some plant extracts, such as those from *Tetrapleura tetraptera* and *Piper* species [89] display significant activity against *Biomphalaria glabrata*. Similarly, aqueous and ethyl acetate crude extracts of *Glinus lotoides* fruit [91] and methanolic extracts from fresh *Solanum aculeastrum* root bark and berries [92] show molluscicidal activity against *Biomphalaria pfeifferi*. Crude camellia and mangosteen extracts are effective molluscicides for controlling *Bithynia siamensis goniomphalos* [93]. *Punica granatum* and *Canna indica* may have potent effects against *Lymnaea acuminata*, and the concentrations used to kill snails are non-toxic to fish [94]. Linalool, derived from *Cinnamomum camphora*, shows molluscicidal activity against *O. hupensis* and may work by damaging the gills and hepatopancreas [88]. Products from *Hypericum* species hexane extracts may be used as potential molluscicides to control *Radix peregra* snails [95].

Biological control is another method used to reduce snail populations and influence the transmission of SBPDs. In Senegal, field trials have demonstrated that water stocked with predatory prawns (*Macrobrachium vollenhoveni*) led to fewer infected snails and reduced schistosomiasis transmission in villages [96]. A laboratory experiment showed that predatory prawns prefer to consume snails infected with schistosomes, and young and growing prawns kill snails most efficiently [97]. The water bug, *Sphaerodema urinator*, shares a common habitat with freshwater snails and has been used to control host snails that transmit schistosomiasis. One study indicated that *S. urinator* may be an effective biological agent as a predator of the intermediate hosts of *Schistosoma* in water [98]. The black carp, *Mylopharyngodon piceus*, is a noteworthy predator of snails that are intermediate hosts of *C. sinensis* and *O. viverrini*. Investigations showed that black carp can decrease snail population densities under both semi-field and field conditions and have been used successfully as biological controls in different regions of the world [99]. Although the potential of biologically controlling freshwater snails has received recent attention, it may negatively impact human health. However, when biological control is successful, it is mutually beneficial to both humans and nature [96].

## Conclusions

SBPDs, including most trematodiasis diseases (clonorchiasis, fascioliasis, fasciolopsiasis, opisthorchiasis, paragonimiasis and schistosomiasis) and some nematodiasis diseases (e.g., angiostrongyliasis) with an expanding geographical distribution, remain highly prevalent worldwide and have substantial deleterious impacts on human health, predominantly in tropical and sub-tropical areas. Consequently, breaking the disease transmission cycle by controlling host snail populations is an alternative method of reducing the spread of such diseases due to the lack of clinically effective SBPD vaccines and potential parasite resistance to the currently available anthelmintic drugs.

Compared with physical and synthesized chemical molluscicide control methods, plant-derived molluscicides are more environmentally friendly, less toxic and are less likely to cause snails to develop resistance, suggesting a promising novel method of reducing endemic snail populations. In addition, comprehensive molecular epidemiology studies, an understanding of the ecology of medically important snails and further insights into snail-parasite interactions, particularly those based on large-scale data mining of genomic snail datasets, are necessary to identify specific or key molecules involved in snail survival, metabolism and development. These molecules could be potential targets for natural molluscicides, which could be developed as novel and effective treatment and control strategies against SBPDs.

## Additional file

Additional file 1: Multilingual abstracts in the five official working languages of the United Nations. (PDF 352 kb)

## Abbreviations

Ac: *Angiostrongylus cantonensis*
Cs: *Clonorchis sinensis*
EM: Eosinophilic meningitis
Fb: *Fasciolopsis buski*
Fh: *Fasciola hepatica*
L1: First-stage larvae
L2: Second-stage larvae
L3: Third-stage larvae
NaPCP: Sodium pentachlorophenate
OA: Ocular angiostrongyliasis
Of: *Opisthorchis felineus*
Ov: *Opisthorchis viverrini*
Pw: *Paragonimus westermani*
SBPDs: Snail-borne parasitic diseases
Sh: *Schistosoma haematobium*
Si: *Schistosoma intercalatum*
Sj: *Schistosoma japonicum*
Smal: *Schistosoma malayensis*
Sman: *Schistosoma mansoni*
Smek: *Schistosoma mekongi*
WPN: niclosamide ethanolamine salt
