# Supplementary material for: Snail-borne parasitic diseases: an update on global epidemiological distribution, transmission interruption and control methods
Source: Infect Dis Poverty. 2018 Apr 9;7:28. doi: 10.1186/s40249-018-0414-7 (PMC5890347; doi:10.1186/s40249-018-0414-7)
Supplement: Supplementary file 1 — Multilingual abstracts in the five official working languages of the United Nations. (PDF 352 kb) [file 40249_2018_414_MOESM1_ESM.pdf]

Translation of the abstract into the five official working languages of the United Nations

الأمراض الطفيلية التي ينقلها الحلزون: تحديث بشأن التوزيع العالمي للأوبئة، وأماكن انقطاع انتقالها وطرق التحكم فيها

شياو-تينغ لو، كيو-يون غو، يانين ليمبانون، زونغ-داو وو، لان غوي سونج، كامولنيتر أوكانوراك، زهي يو لف

الملخص

الخلفية: تشكل الأمراض الطفيلية المنقولة بواسطة الحلزون، مثل داء ديدان Angiostrongylus الاسطوانية ، وداء متفرعات الخصية، وداء المتورقات، وداء المتوارقات، وداء الديدان متأخرات الخصية، وداء جانبية المناسل والبلهارسيا خطراً على صحة الإنسان وتسبب مشاكل اجتماعية واقتصادية رئيسية في العديد من البلدان الاستوائية وشبه الاستوائية. في هذا الاستعراض نلخص الأدوار الأساسية للقواقع في دورات حياة الطفيليات التي تستضيفها ، ومظاهرها السريرية وتوزيعات الأمراض بالإضافة إلى أساليب مكافحة الحلزون.

المحتوى الرئيسي: لدى الحلزونات (القواقع) أربعة أدوار في دورات حياة الطفيليات التي تستضيفها: كمضيف وسيط مصاب بيرقات المرحلة الأولى، ومضيف وسيط وحيد مصاب باليرقات المهدبة، وأول مضيف وسيط يبتلع بيض الطفيليات ويهضمها، وأول مضيف وسيط توغلت به اليرقات المهدبة مع أو بدون المضيف الوسيط الثاني كحيوان مائي. الأمراض الطفيلية التي ينقلها الحلزون تستهدف العديد من الأجهزة، مثل الرئتين، والكبد، والمسالك الصفراوية، والأمعاء، والدماغ والكليتين، مما يؤدي إلى فرط نشاط الاستجابات المناعية والسرطانات، وفشل العضو، والعقم بل والموت. تحتوي البلدان النامية في أفريقيا وآسيا وأمريكا اللاتينية على أعلى حالات إصابة بهذه الأمراض، في حين تطورت بعض الطفيليات المتوطنة إلى أوبئة في جميع أنحاء العالم من خلال انتشار القواقع على الصعيد العالمي. وقد تم استخدام الأساليب الفيزيائية والكيميائية والبيولوجية للسيطرة على تجمعات الحلزون المضيف للوقاية من الأمراض.

الاستنتاجات: في هذا الاستعراض لخصنا أدوار القواقع في دورات حياة الطفيليات التي تستضيفها، والتوزيع في جميع أنحاء العالم للقواقع ناقلة الطفيل، ودراسة الأوبئة وطريقة تطور الأمراض الطفيلية التي ينقلها الحلزون والإجراءات الحالية للتحكم في القواقع والتي سوف تسهم في زيادة فهم علاقة الطفيل بالحلزون والاستراتيجيات الجديدة للسيطرة على الأمراض الطفيلية التي ينقلها الحلزون.

Translated from English version into Arabic by Free Bird and Tarek Salem, through

TRANSLATORS WITHOUT BORDERS

螺传寄生虫病的全球分布、传播阻断与控制措施

卢晓婷，顾秋云，Yanin Limpanont，宋兰桂，吴忠道，Kamolnetr Okanurak，吕志跃

摘要

引言：在许多热带和亚热带国家中，螺传寄生虫病（如广州管圆线虫病、华支睾吸虫病、肝片形吸虫病、姜片虫病、后睾吸虫病、并殖吸虫病和血吸虫病等）仍然威胁人类健康并引起重大的社会经济问题。本综述概述了螺在寄生虫生活史中的重要地位、螺传寄生虫病的临床表现与全球分布、以及控螺措施。

正文：螺可在寄生虫生活史中扮演 4 种角色：（1）作为中间宿主，被第一期幼虫感染；（2）作为唯一的中间宿主，被毛蚴感染；（3）作为第一中间宿主，吞食虫卵；（4）作为第一中间宿主，被毛蚴侵入感染，且以水生动物为第二中间宿主或水生植物为传播媒介。螺传寄生虫

感染可损害多种器官，如肺脏、肝脏、胆道、肠道、大脑和肾脏等，导致宿主免疫应答过高、肿瘤、器官衰竭、不育甚至死亡。非洲、亚洲和拉丁美洲的发展中国家中螺传寄生虫病的发病率最高，但由于螺在全球范围内的扩散，原先一些局部流行的寄生虫病已呈全球分布。利用物理、化学和生物方法可有效控制螺的数量，以预防螺传寄生虫病的发生。
结论：本文概述了螺在寄生虫生活史中的作用、传播寄生虫病的螺的世界分布、螺传寄生虫病的流行病学和发病机制，以及现有螺类控制措施。这些知识将有助于进一步了解螺与寄生虫之间的相互关系，并探讨螺传寄生虫病的新防控策略。

Translated from English version into Arabic by Xiao-Ting Lu

Les maladies parasitaires transmises par les escargots : une mise à jour sur la distribution épidémiologique mondiale, l'interruption de la transmission et les méthodes de contrôle

Xiao-Ting Lu, Qiu-Yun Gu, Yanin Limpanont, Lan-Gui Song, Zhong-Dao Wu, Kamolnetr Okanurak, Yue-Zhi Lv

Résumé

Contexte : les maladies parasitaires transmises par les escargots, telles que l'angiostrongylose, la clonorchiase, la fasciolase, la fasciolopsiase, l'opisthorchiose, la paragonimose et la schistosomiase, posent des risques pour la santé humaine et causent de graves problèmes socio-économiques dans de nombreux pays tropicaux et subtropicaux. Dans cette revue, nous résumons les rôles principaux des escargots dans les cycles de vie des parasites qu'ils hébergent, leurs manifestations cliniques et la distribution des maladies, ainsi que les méthodes de contrôle des escargots.
Partie principale : les escargots ont quatre rôles dans les cycles de vie des parasites qu'ils hébergent : comme un hôte intermédiaire contaminé par la larve à son premier stade, comme le seul hôte intermédiaire contaminé par le miracidium, comme le premier hôte intermédiaire qui ingère les oeufs des parasites et comme le premier hôte intermédiaire pénétré par le miracidium, que le second hôte intermédiaire soit un animal aquatique ou non. Les maladies parasitaires transmises par les escargots ciblent de nombreux organes, tels que les poumons, le foie, les voies biliaires, les intestins, le cerveau et les reins, provoquant des réactions immunitaires hyperactives, des cancers, des insuffisances au niveau de certains organes, l'infertilité et même la mort. Les pays en voie de développement en Afrique, Asie et Amérique latine ont les taux les plus élevés de ces maladies, alors que certains parasites endémiques se sont transformés en épidémies dans le monde entier par le biais de la propagation mondiale des escargots. Des méthodes physiques, chimiques et biologiques sont utilisées pour contrôler les populations d'escargots hôtes afin d'éviter les maladies.
Conclusions : dans cette revue, nous résumons les rôles des escargots dans les cycles de vie des parasites qu'ils hébergent, la distribution mondiale des escargots transmetteurs de parasites, l'épidémiologie et la pathogenèse des maladies parasitaires transmises par les escargots, et les mesures de contrôle d'escargots en place, ce qui contribuera à une meilleure compréhension de la relation escargot-parasite et à de nouvelles stratégies pour contrôler les maladies parasitaires transmises par les escargots.

Translated from English version into French by Maud S and Frank, through

Паразитарные заболевания, распространяемые улитками: последняя информация о глобальном эпидемиологическом распространении, методах предотвращения и ограничения передачи

Сяо-Тин Лу, Цю-Юнь Гу, Янин Лимпапонт, Песнь Лан-Гуи, Чжун-Дао У, Камолнетр Оканурак, Чжи-Юй Lv

Аннотация

Вводная информация: Паразитарные заболевания, распространяемые улитками (такие как ангиостронгилиоз, клонорхиоз, фасциолез, фасциолопсиоз, описторхоз, парагонимиоз и шистосомоз), создают риск для здоровья человека и вызывают серьезные социально-экономические проблемы во многих тропических и субтропических странах. В данной статье обобщаются основные роли улиток в жизненном цикле паразитов, которых они переносят, клинические проявления болезней и их распространение, а также методы борьбы с улитками.

Основная часть: Существует четыре роли, которые улитки могут исполнять в жизненном цикле переносимых ими паразитов: промежуточный хозяин, заражённый личинками первой стадии; единственный промежуточный хозяин, заражённый мирацидиями; первый промежуточный хозяин, поглотивший яйца паразита; первый промежуточный хозяин мирацидий со вторым промежуточным хозяином-водным животным или без него. Паразитарные заболевания, распространяемые улитками, поражают многие органы, такие как легкие, печень, желчный тракт, кишечник, мозг и почки, что приводит к гиперактивным иммунным реакциям, раку, функциональной недостаточности органов, бесплодию и даже смерти. Самую высокую заболеваемость этими болезнями имеют развивающиеся страны Африки, Азии и Латинской Америки. Вместе с тем, вследствие глобального распространения улиток некоторые эндемичные паразиты распространились по всему миру. Для предотвращения болезней были введены физические, химические и биологические методы контроля популяций улиток.

Выводы: В данной статье даётся обобщённое представление о роли улиток в жизненном цикле переносимых ими паразитов, о глобальном распространении улиток-переносчиков, эпидемиологии и патогенезе паразитарных заболеваний, передаваемых улитками, и о текущих мерах контроля популяции улиток, что будет способствовать более глубокому пониманию взаимоотношений между улиткой и паразитом и появлению новых стратегий борьбы с паразитарными заболеваниями, распространяемыми улитками.

Translated from English version into Russian by Tatiana Kary and Elena Alieva, through

Enfermedades parasitarias transmitidas por el caracol: una actualización sobre métodos de control, interrupción de la transmisión y distribución epidemiológica mundial

Xiao-Ting Lu, Qiu-Yun Gu, Yanin Limpanont, Lan-Gui Song, Zhong-Dao Wu, Kamolnetr Okanurak, Zhi-Yue Lv

Resumen

Antecedentes: las enfermedades parasitarias transmitidas por el caracol, tales como la angiostrongiliasis, clonorquiasis, fascioliasis, fasciolopsiasis, opistorquiasis, paragonimiasis y esquistosomiasis, suponen un riesgo para la salud humana y causan grandes problemas socioeconómicos en muchos países tropicales y subtropicales. En este resumen analizamos las principales funciones de los caracoles en los ciclos de vida de los parásitos que alojan, sus manifestaciones clínicas y distribución de enfermedades, así como los métodos de control de caracoles.

Texto principal: Los caracoles desempeñan cuatro funciones en los ciclos de vida de los parásitos que alojan: como hospedador intermediario infectado por una larva en primera fase, como el único hospedador intermediario infectado por miracidios, como el primer hospedador intermediario que ingiere los huevos parasitarios, y como el primer hospedador intermediario penetrado por miracidios con o sin que el segundo hospedador intermediario sea un animal acuático. Las enfermedades parasitarias transmitidas por el caracol afectan a varios órganos, como los pulmones, el hígado, el tracto biliario, los intestinos, el cerebro y los riñones; esto provoca una respuesta inmune hiperactiva, cáncer, insuficiencia orgánica, infertilidad e incluso la muerte. Los países en desarrollo de África, Asia y América Latina sufren el mayor número de incidencias de estas enfermedades, mientras que algunos parásitos endémicos se han convertido en epidemias mundiales mediante la propagación a nivel global de caracoles. Para prevenir enfermedades, se han introducido métodos físicos, químicos y biológicos para controlar las poblaciones de caracoles hospedadores.

Conclusiones: En este resumen, hemos analizado las funciones de los caracoles en los ciclos de vida de los parásitos que hospedan, la distribución mundial de caracoles transmisores de parásitos, la epidemiología y patogénesis de las enfermedades parasitarias transmitidas por caracoles, y las medidas existentes de control de caracoles, que contribuirán a una mayor comprensión de la relación entre caracoles y parásitos, y las nuevas estrategias para el control de las enfermedades parasitarias transmitidas por caracoles.

Translated from English version into Spanish by Ana Tranorte and Mayra Leon , through
